# P-351. Hospital associated outbreak of Crimean-Congo Hemorrhagic Fever, Dushanbe, Tajikistan, 2023

**DOI:** 10.1093/ofid/ofae631.552

**Published:** 2025-01-29

**Authors:** Rajabali Sharifov, Roberta Horth, Zulfiya H Tilloeva, Navruz Jafarov, Dilyara Nabirova

**Affiliations:** Central Asia Region FETP, Dushanbe, Dushanbe, Tajikistan; US Centers for Disease Control and Prevention, Dulles, Virginia; City Disinfection Station, Dushanbe, Republic of Tajikistan, Dushanbe, Dushanbe, Tajikistan; Ministry of Health, Dushanbe, Dushanbe, Tajikistan; CDC Central Asia office, Almaty, Almaty, Kazakhstan

## Abstract

**Background:**

On July 1, 2023, a surgeon (Patient B) in Dushanbe, Tajikistan, died of acute febrile illness with hemorrhaging. Patient B had been hospitalized with acute respiratory distress four days before. Clinicians suspected Crimean-Congo Hemorrhagic Fever (CCHF). CCHF is a rare tick-borne disease. Tajikistan records just 1-6 cases annually. Patient B had no known exposures to ticks. We aimed to identify source and additional cases, and determine source.

**Figure 1.**
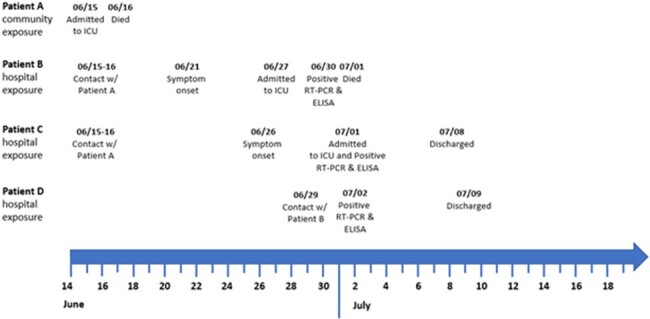
Timeline of CCHF Hospital Outbreak, Dushanbe, Tajikistan, 2023

**Methods:**

We conducted an outbreak investigation on July 1st. A confirmed case was defined as a person with a positive anti-CCHF IgM ELISA or RT-PCR and an epidemiological link. We reviewed medical charts to find contacts, defined as people who had been in direct contact with or had exposure to bodily fluids from any confirmed case in the last 21 days. We interviewed living cases and contacts and collected blood for testing. We also conducted an infection control assessment.

**Figure 2.**
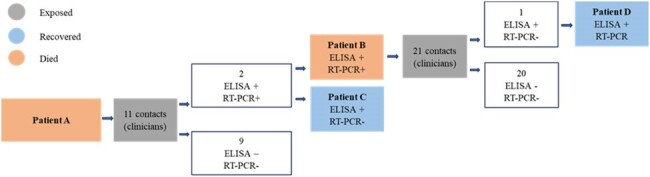
Cases and contacts in CCHF Hospital Outbreak, Dushanbe, Tajikistan, 2023

**Results:**

A hospitalized adult (Patient A) had died of internal bleeding with extensive peritonitis (Figure 1) on June 16th. Six days later, a physician (Patient B) who had performed surgery on Patient A developed fever. Another six days later, he was hospitalized with hemorrhagic fever and diagnosed with CCHF within 3 days. Another physician (Patient C) who had performed surgery on Patient A was diagnosed with CCHF the following day. A laboratory assistant (Patient D) who had given a direct transfusion to Patient B was also diagnosed with CCHF the next day. Case-fatality rate was 25% (Table 1). We identified 29 healthcare providers who had contact to Patients A-D. All were tested, isolated and given oral Ribavirin 400 mg for 3 days as prophylaxis. None became ill (Figure 2). Interviews with hospital staff revealed infection control gaps, including lack of personal protective equipment during surgery and invasive procedures.

**Table 1.**
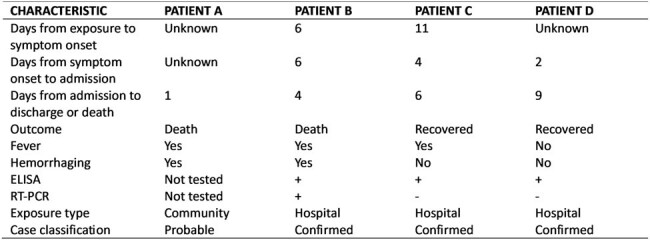
Characteristics of CCHF cases in a hospital outbreak in Dushanbe, Tajikistan, 2023

**Conclusion:**

Misdiagnosis and infection control gaps contributed to transmission of CCHF virus within the hospital. Direct blood transfusion is not a recommended practice, but performed due to lack of resources. Trainings were provided on infection prevention and control, and on CCHF diagnosis and care. No additional cases were detected.

**Disclosures:**

**All Authors**: No reported disclosures

